# Factors affecting awareness of myocardial infarction symptoms among the general public in Korea

**DOI:** 10.4178/epih.e2020032

**Published:** 2020-05-18

**Authors:** Kyong Sil Park

**Affiliations:** School of Nursing, Cheju Halla University, Jeju, Korea

**Keywords:** Myocardial infarction, Cardiovascular diseases, Awareness, Knowledge, Korea Community Health Survey

## Abstract

**OBJECTIVES:**

We aimed to determine the level of awareness of myocardial infarction (MI) symptoms among the general public in Korea and identify factors affecting awareness of MI symptoms using data from the 2017 Korea Community Health Survey (KCHS).

**METHODS:**

This is a cross-sectional study using KCHS data. Based on five questions about MI symptoms, participants were divided into an awareness group (replied ‘yes’ to all five questions) and an unawareness group (replied ‘no’ or ‘not sure’ to at least one of five questions) for analysis.

**RESULTS:**

Of a total of 228,281 participants, 42.4% were aware of MI symptoms. There was a high level of awareness of chest pain and shortness of breath, but a low level of awareness of gastrointestinal symptoms and pain in the arm, shoulder, jaw, neck, and back. While women had a higher level of overall awareness relative to men, they showed a lower level of awareness regarding chest pain and discomfort. The factors affecting awareness of MI symptoms were gender, age, education level, occupation, smoking, drinking, physical inactivity, and cardiovascular disease risk factors.

**CONCLUSIONS:**

In order to enhance awareness of MI symptoms among the general population, appropriate education and promotion efforts must be implemented based on gender, age, education level, and occupation. Moreover, active efforts by the government, educational institutions, and medical institutions are necessary to improve awareness of both typical and atypical MI symptoms. Furthermore, health policies to promote reduced smoking and drinking and increased physical activity, as well as continuous monitoring and management of individuals with cardiovascular disease risk factors, are required.

## INTRODUCTION

Deaths attributable to heart disease occurred at a rate of 62.4 per 100,000 people in 2018, which has steadily increased from 43.4 per 100,000 people in 2008. In particular, the rate of deaths due to ischemic heart disease, such as myocardial infarction (MI) and angina, was 30.9, accounting for approximately 50% of deaths due to heart disease [1]. While the rate of deaths due to cerebrovascular diseases is on the decline, heart disease mortality continues to increase [1] and the socioeconomic burden of this disease is high. MI occurs when the heart’s coronary artery is completely blocked by a blood clot, resulting in necrosis of the heart muscle. Since it can cause complications like heart failure, arrhythmia, disability, and death, swift response and treatment immediately following MI is the most critical determinant of prognosis [2].

In 2017, the awareness of acute MI symptoms was 45.5% in general public [3]. In addition, after the onset of symptoms, only 47.7% of patients arrived at the hospital within three hours—considered to be the critical period for intervention for cerebrovascular disease [4]—showing that awareness of and initial response to heart disease is still poor. In order to improve the healthcare system’s capability for early intervention, it is important for individuals be aware of the usual symptoms and how to respond so that patients with heart disease and pre-existing conditions as well as community-dwelling individuals can receive proper treatment within the critical period in the event of an MI [3].

The treatment-seeking time of MI patients, from initial symptom recognition to hospital visit, was on average 13 hours for men and 20 hours for women, who displayed more passive behavior and attitudes toward symptoms than men [5]. Regarding MI symptoms, men complained of typical symptoms including chest pain and chills, whereas women complained of atypical symptoms like pain in the back, jaw, and neck, upper abdominal discomfort, dyspnea, fatigue, and loss of appetite [5-7]. In particular, Korean MI patients mistook the cause of their pain as ‘indigestion or gastrointestinal problems’ instead of the heart [5]. The low level of awareness among the general population and gender-specific perceptions and attitudes about MI symptoms cause delays in seeking treatment, and hinder proper treatment within the critical period. For a swift response in the occurrence of an MI, it is necessary to determine the level of awareness among the general population and investigate whether they are displaying proper treatment-seeking behavior.

The factors that affect the degree of awareness of MI symptoms are gender, age, race, education level, income, occupation, chronic illness, smoking, drinking, physical activity, an acquaintance’s experience with MI, knowledge of MI, and contact with promotional materials [8-19]. Although there have been studies on awareness among Koreans, these studies have limitations such as being conducted in a restricted area with a small number of participants or neglecting the behavioral and metabolic risk factors of cardiovascular disease (CVD) in favor of solely socioeconomic factors.

Therefore, we aim to identify the level of awareness of MI symptoms and determine factors affecting awareness among the general population using data from a large-scale local community survey including five items on MI symptoms.

## MATERIALS AND METHODS

### Research design

This is a cross-sectional study using data from the 2017 Korea Community Health Survey (KCHS) to identify the degree of and factors affecting awareness of MI symptoms among the general population based on gender.

### Study population

We used raw data from the 2017 KCHS. Of the 228,381 participants assessed in the raw data, we excluded 100 individuals who replied ‘refuse to respond’ to the questions on MI symptoms. A final sample of 228,281 individuals were selected as study participants.

### Measurements

Demographic information including gender, age, education level, income level, marital status, occupation, monthly household income, and residential area was collected. Occupation was categorized into professional/manager/clerk (i.e., managers, professionals and related workers, clerks), sales/service/manual (i.e., service workers, sales workers, agricultural/forestry/fishery workers, craft and related trades workers, equipment/machine operating and assembling workers, elementary workers, armed forces), and unemployed/housewives (i.e., unemployed, housewives, students). CVD, hypertension, diabetes, and dyslipidemia were considered to be present if the participant responded ‘yes’ to ‘doctor’s diagnosis’ or ‘currently receiving treatment’ regarding the respective health conditions.

To assess awareness of MI symptoms, participants were asked about their awareness of the five major symptoms of MI as presented by the United States Centers for Disease Control and Prevention (CDC) [20]. Participants were asked, ‘Which do you think are MI symptoms?’ and responded to the following options: (1) pain or discomfort in the jaw, neck, or back, (2) weakness, dizziness, nausea, and/or cold sweat, (3) pain, pressure, or squeezing in the chest, (4) sudden pain or discomfort in the arm/shoulder, and (5) sudden shortness of breath. Participants were asked to respond either ‘yes’ or ‘no.’ Participants who replied ‘not sure’ to an MI symptom were considered to have no knowledge of MI and the answer was re-coded to ‘no.’ Participants who replied ‘refuse to respond’ were processed as missing and excluded from participant selection. Participants who replied ‘yes’ to all were assigned to the ‘awareness group’ and those who replied ‘no’ to at least one of the five were assigned to the ‘unawareness group’ [9,10,14].

### Statistical analysis

We used SPSS version 20 (IBM Co., Armonk, NY, USA) for data analysis. Nominal variables are presented as the number and percentage of cases, and continuous variables are presented as the mean and standard deviation. We performed a chi-squared test to determine the awareness of MI symptoms and a multivariate logistic regression to identify the factors affecting odds of belonging to the awareness or the unawareness groups. We set the significance level at p<0.05.

### Ethics statement

Since this study was conducted using KCHS data, ethics approval was not required. We applied through the KCHS website for approval and received raw data excluding personal information from KCHS.

## RESULTS

Regarding demographic characteristics, 44.9% were men and 55.1% were women, and 39.2% were aged 60 years or older. Of the total participants, 17.5% were current smokers and 67.2% were current drinkers. Of the total participants, 66.2% of physical activity, and 27.4% had hypertension, 11.0% had diabetes, 17.4% had dyslipidemia, and 27.4% had a body mass index (BMI) of ≥ 25 kg/m^2^ (Table 1).

Of the total participants, 11.8% failed to recognize any of the five MI symptoms and 42.4% recognized all of them. In the unawareness group, 20.5% failed to recognize any of the five symptoms and 28.2% recognized four out of five symptoms (Table 2).

We compared the five symptoms of MI and found that the level of awareness was 83.0% for ‘pain, pressure, or squeezing in the chest,’ 78.2% for ‘sudden shortness of breath,’ 69.2% for ‘weakness, dizziness, nausea, and/or cold sweat,’ 63.3% for ‘pain or discomfort in the jaw, neck, or back,’ and 53.8% for ‘sudden pain or discomfort in the arm/shoulder.’ Men showed higher awareness of the symptom of ‘pain, pressure, or squeezing in the chest’ than women, but women showed higher awareness of the other four symptoms relative to men. In terms of age, participants aged 40-59 years showed high awareness of all five symptoms, while those aged ≥ 60 years showed the lowest awareness. In terms of education level, participants with a college education or greater showed higher awareness of all five symptoms than participants with a high school-level education. Married participants showed higher awareness for all five symptoms than unmarried participants. In terms of occupation, participants in the professional/manager/clerk category showed the highest awareness, while unemployed/housewives showed the lowest. The higher the monthly household income, the higher the awareness for each symptom. Smokers and drinkers showed low awareness, whereas non-smokers and non-drinkers showed high awareness. Participants with good physical activity showed high awareness. Participants who had all of the CVD risk factors (i.e., hypertension, diabetes, dyslipidemia, and obesity) showed the highest awareness of the symptoms (Table 3).

We compared the expected treatment-seeking behavior in the event of MI and found that 85.6% and 81.3% of the participants in the awareness and unawareness groups, respectively, replied ‘call 119,’ showing high treatment-seeking behavior (Table 4).

We performed a multivariate logistic regression analysis to identify the demographic and health-related factors affecting awareness of MI symptoms and found that women (odds ratio [OR], 1.03; 95% confidence interval [CI], 1.01 to 1.06; p= 0.010), age of 40-59 years (OR, 1.32; 95% CI, 1.29 to 1.35; p<0.001), college education (OR, 1.31; 95% CI, 1.28 to 1.33; p<0.001), unemployed/housewives (OR, 0.81; 95% CI, 0.79 to 0.84; p<0.001), current smokers (OR, 0.84; 95% CI, 0.82 to 0.86; p<0.001), current drinkers (OR, 0.98; 95% CI, 0.95 to 1.00; p<0.05), participants performing at least 150 minutes of moderate-intensity physical activity or 75 minutes of high-intensity physical activity per week (OR, 1.03; 95% CI, 1.01 to 1.04; p<0.01), and participants with all four CVD risk factors (OR, 1.20; 95% CI, 1.11 to 1.29; p<0.001) were the factors affecting the recognition of acute MI symptoms (Table 5).

## DISCUSSION

For proper emergency treatment within the critical period after onset of MI, it is necessary to be well-aware of the symptoms of MI [3]. However, 11.8% of participants failed to recognize any of the five MI symptoms. Conversely, 42.4% of participants were in the awareness group, meaning they recognized all five symptoms. According to a study conducted in Korea in 2010, awareness of all five symptoms were 7.1% [9], 10.9% [10], which increased for about 7 years. This may be a result of continuous education about early symptoms and promotional activities that had occurred since 2008 after the designation of a regional cerebrovascular disease center [4]. However, the proportion of the general public in Korea that is aware of all five symptoms is relatively low compared to 53.0% in the United States [13], and thus a management plan is necessary to enhance the level of awareness. Once an improved perception of MI symptoms is established among Koreans, then proper treatment within the critical period of MI will be possible and the socioeconomic burden of the disease can be greatly reduced.

In this study, participants were well-aware that the symptom of ‘pain, pressure, or squeezing in the chest’ is a symptom of MI, but a high percentage failed to recognize ‘pain or discomfort in the arm/shoulder’ and ‘pain or discomfort in the jaw, neck, or back’ as MI symptoms. Compared to the results of the 2010 study in Korea [9,10], overall awareness has increased, but the level of awareness for each symptom is still low. In other words, chest pain is recognized as a typical MI symptom, but the symptom of pain radiating to the arm, shoulder, jaw, and neck showed a low recognition rate of 53.8%. In the United States [13], the level of awareness for ‘pain or discomfort in the arm/shoulder’ was 85.7%, showing a significant awareness gap between the United States and Korea. While the level of awareness of the five MI symptoms were generally high overseas [13], in Korea, awareness of symptoms other than chest pain and shortness of breath was low. Therefore, education and promotion must be focused on those symptoms that have low levels of awareness rather than the well-known MI symptoms.

We confirmed gender as a predictor of MI symptoms awareness. While women showed higher overall awareness of MI symptoms than men, men showed higher awareness of the symptom of ‘pain, pressure, or squeezing in the chest’ than women. Given that women showed higher awareness for all five major symptoms of MI in study from the United States [14], it can be inferred that for an ordinary Korean man, chest pain and discomfort are recognized as the typical symptom of MI. This suggests that when other MI symptoms arise, especially when atypical MI symptoms develop in a women family member, timely CVD intervention and treatment will be less likely. Therefore, it is necessary to educate men on MI symptoms other than the typical symptoms.

Age was a major predictor of MI symptoms awareness, such that the group aged 40-59 years had higher awareness than other age groups. Since middle-aged people aged 40-59 years are at a high risk of experiencing an MI [17], they are commonly exposed to disease-related information and display a high level of awareness of symptoms. On the other hand, young adults aged 19-39 years tend to lack the time and need for MI education [21] and thus had low levels of symptom recognition. Finally, individuals aged 60 years or older lack understanding of MI symptoms, likely due to aging [5].

Participants with a high socioeconomic status (SES), comprising education level and occupation, had a high-level of awareness of MI symptoms. In a previous study, education-level and occupation were associated with MI awareness [8-19]. Individuals with a high SES are under favorable conditions for actively receiving information about MI and carrying it out. On the other hand, individuals with a low SES have limited access to information or unfavorable conditions [22], which may affect the level of awareness. Therefore, to reduce health inequality based on SES, it is necessary to focus on individuals with a low education-level, sales/service/manual workers, and unemployed/housewives and to perform promotion and education tailored to them. In particular, individuals in their 20s and 30s have high-levels of Internet usage and individuals in their 60s or above can improve their awareness by using a health center [11], and thus we must employ various methods for effective promotion and education.

CVD risk factors including smoking, drinking, and physical inactivity were confirmed to be factors affecting MI symptoms awareness. Participants who smoke or drink showed low MI symptoms awareness. Previous studies have reported that smoking and heavy drinking were strongly correlated with knowledge of CVD [11,12,19]. Behaviors that are harmful to health such as smoking and drinking arise from a lack of overall awareness of health problems, and this lack of general health awareness may have caused the low level of awareness of MI symptoms. Participants who perform at least 150 minutes of moderate-intensity physical activity or 75 minutes of high-intensity physical activity per week had high MI symptoms awareness. Although research is lacking on the association between physical activity and MI awareness, a recent study reported that regular exercise is associated with MI awareness [12]. While smoking, drinking, and physical inactivity are behavioral risk factors for CVD [23], they are also associated with recognition of CVD symptoms. Since a change in health awareness is the major driver behind changes in health behaviors [24], we need to pay close attention to this and make efforts to enhance awareness of MI symptoms together with health behaviors like smoking, drinking, and physical inactivity.

Participants with at least three CVD risk factors including hypertension, diabetes, dyslipidemia, and obesity showed high MI symptoms awareness. Individuals with many risk factors have high MI symptoms awareness because they are aware that these risk factors are the typical CVD risks. On the other hand, participants with two or fewer risk factors had low-level or insignificant-level of awareness. This can be explained by a previous finding that participants with hypertension and dyslipidemia show high awareness, whereas those with diabetes and obesity show low-level or insignificant-level of awareness [11,12]. The low MI symptoms awareness in participants with diabetes and obesity may have been affected by the number of risk factors. However, to verify this relationship, further research on the association between typical CVD risk factors and MI symptoms awareness is required.

In this study, we targeted the general population, not MI patients, to investigate awareness of the five typical MI symptoms and identify factors that affect awareness. This research is meaningful in that it can be used to guide educational interventions for enhancing recognition of and response to early MI symptoms. Since we used representative data, the results may be generalized to the entire population. However, a limitation is that this is a cross-sectional study based on a self-report survey at a single time point, so it cannot be used to establish a causal relationship; another limitation is that missing data were excluded.

In order to enhance awareness of MI symptoms among the general population in Korea, education and promotion must be conducted with considerations to gender, age, education level, and occupation. Moreover, active engagement by the government, educational institutions, and medical institutions is required for awareness of both typical and atypical MI symptoms. We also need health policy management plans to promote non-smoking, non-drinking, and physical activity, as well as consistent follow-up and management of participants with multiple CVD risk factors. These efforts will increase awareness of MI symptoms and shorten the treatment-seeking time of MI patients to improve their quality of life, reduce the socioeconomic burden of the disease, and positively influence public health policies.

## Figures and Tables

**Table 1.** Socio-demographic and health-related characteristics (n=228,281)

Characteristics	Category	n (%)
Gender	Men	102,431 (44.9)
	Women	125,850 (55.1)
Age (yr)	19-39	54,203 (23.7)
	40-59	84,600 (37.1)
	≥60	89,478 (39.2)
Education	High school	158,978 (69.6)
	≥College	69,303 (30.4)
Marital status	Married	192,864 (84.6)
	Unmarried/divorced/widowed	35,167 (15.4)
Occupation	Professional/manager/clerk	44,952 (19.7)
	Sales/service/manual	99,390 (43.6)
	Unemployed/housewives	83,674 (36.7)
Monthly household income (10,000 Korean won)	<200	81,663 (36.1)
	200-399	74,647 (33.0)
	≥400	69,898 (30.9)
Residential area	Urban	109,362 (47.9)
	Rural	118,919 (52.1)
Smoking	Current smoker	39,965 (17.5)
	Ex-smoker	42,041 (18.4)
	Never	146,268 (64.1)
Drinking	Current drinker	153,441 (67.2)
	Ex-drinker	32,887 (14.4)
	Never	41,940 (18.4)
Physical activity	Bad	150,977 (66.2)
	Good^1^	77,191 (33.8)
CVD risk factors	Hypertension	62,500 (27.4)
	Diabetes mellitus	25,200 (11.0)
	Dyslipidemia	39,775 (17.4)
	BMI ≥25 kg/m^2^	60,037 (27.4)

CVD, cardiovascular disease; BMI, body mass index.

1 At least 150 minutes of moderate-intensity physical activity or 75 minutes of vigorous-intensity physical activity throughout the week.

**Table 2.** Awareness of myocardial infarction symptoms

Category	Total (n=228,281)	Awareness (n=96,783)	Unawareness (n=131,498)
None	26,985 (11.8)	0 (0.0)	26,985 (20.5)
1	9,822 (4.3)	0 (0.0)	9,822 (7.5)
2	21,592 (9.5)	0 (0.0)	21,592 (16.4)
3	35,975 (15.8)	0 (0.0)	35,975 (27.4)
4	37,124 (16.3)	0 (0.0)	37,124 (28.2)
5	96,783 (42.4)	96,783 (100)	0 (0.0)

Values are presented as number (%).

**Table 3.** Awareness of each symptom of myocardial infarction (n=228,281)

Characteristics	Category	Pain or discomfort in the jaw, neck, or back	Feeling weak, light-headed, or faint, cold sweat	Chest pain or pressure, squeezing	Pain or discomfort in one or both arms or shoulders	Shortness of breath
Total		63.3	69.2	83.0	53.8	78.2
Gender	Men	62.3	68.6	83.2	53.2	77.5
	Women	64.1	69.7	82.8	54.3	78.8
	p-value	<0.001	<0.001	0.013	<0.001	<0.001
Age (yr)	19-39	62.8	67.4	84.4	52.7	78.5
	40-59	67.7	73.6	88.3	57.8	82.9
	≥60	59.4	66.2	77.1	50.7	73.5
	p-value	<0.001	<0.001	<0.001	<0.001	<0.001
Education	High school	60.8	67.5	80.5	51.9	76.2
	≥College	68.9	73.2	88.8	58.2	82.8
	p-value	<0.001	<0.001	<0.001	<0.001	<0.001
Marital status	Married	64.0	70.0	83.1	54.5	78.6
	Unmarried/divorced/widowed	59.4	65.3	82.3	50.0	76.0
	p-value	<0.001	<0.001	<0.001	<0.001	<0.001
Occupation	Professional/manager/clerk	69.7	73.7	89.2	58.8	83.1
	Sales/service/manual	63.2	69.6	83.3	54.0	78.7
	Unemployed/housewives	60.0	66.4	79.3	51.0	75.0
	p-value	<0.001	<0.001	<0.001	<0.001	<0.001
Monthly household income (10,000 KRW)	<200	58.7	65.3	76.8	49.9	73.1
	200-399	65.0	71.0	85.5	55.7	80.3
	≥400	67.0	72.1	87.7	56.6	82.0
	p-value	<0.001	<0.001	<0.001	<0.001	<0.001
Residential area	Urban	62.9	68.5	83.8	53.5	78.6
	Rural	63.7	69.9	82.2	54.1	77.8
	p-value	<0.001	<0.001	<0.001	0.006	<0.001
Smoking	Current smoker	60.4	67.1	82.4	51.4	76.6
	Ex-smoker	63.1	69.4	83.4	53.8	77.9
	Never	64.1	69.8	83.0	54.5	78.7
	p-value	<0.001	<0.001	<0.001	<0.001	<0.001
Drinking	Current drinker	64.5	70.2	85.2	54.5	79.9
	Ex-drinker	61.6	67.9	80.5	52.8	76.3
	Never	60.2	66.8	77.0	52.3	73.5
	p-value	<0.001	<0.001	<0.001	<0.001	<0.001
Physical activity	Bad	62.4	68.3	82.0	53.4	77.3
	Good^1^	65.0	71.0	85.0	54.6	79.9
	p-value	<0.001	<0.001	<0.001	<0.001	<0.001
CVD risk factors^2^	0	64.0	69.6	83.9	54.4	79.1
	1	62.2	68.2	82.0	52.7	77.0
	2	62.5	69.1	81.8	53.7	77.4
	3	64.1	70.9	82.7	54.2	78.4
	4	65.3	72.2	83.9	56.8	79.3
	p-value	0.003	0.118	<0.001	0.167	<0.001

Values are presented as %. CVD, cardiovascular disease; BMI, body mass index.

1 At least 150 minutes of moderate-intensity physical activity or 75 minutes of vigorous-intensity physical activity throughout the week.

2 CVD risk factors included hypertension, diabetes mellitus, dyslipidemia, and BMI ≥25 kg/m^2^.

**Table 4.** Expected treatment-seeking behavior at myocardial infarction onset

Behaviors	Total (n=227,740)	Awareness (n=96,764)	Unawareness (n=130,976)
Hospital	28,387 (12.5)	10,820 (11.2)	17,567 (13.4)
Oriental hospital	440 (0.2)	162 (0.2)	278 (0.2)
Call 119	189,385 (83.2)	82,849 (85.6)	106,536 (81.3)
Call family	7,979 (3.5)	2,397 (2.5)	5,582 (4.3)
Others	1,549 (0.7)	536 (0.6)	1,013 (0.8)

Values are presented as number (%).

**Table 5.** Predictors of awareness of myocardial infarction symptoms

Characteristics	Category	aOR (95% CI)^1^	p-value
Gender	Men	1.00 (reference)	
	Women	1.03 (1.01, 1.06)	0.010
Age (yr)	19-39	1.00 (reference)	
	40-59	1.32 (1.29, 1.35)	<0.001
	≥60	1.06 (1.04, 1.09)	<0.001
Education	High school	1.00 (reference)	
	≥College	1.31 (1.28, 1.33)	<0.001
Occupation	Professional/manager/clerk	1.00 (reference)	
	Sales/service/manual	0.90 (0.88, 0.93)	<0.001
	Unemployed/housewives	0.81 (0.79, 0.84)	<0.001
Smoking	Never	1.00 (reference)	
	Ex-smoker	0.96 (0.93, 0.99)	0.006
	Current smoker	0.84 (0.82, 0.86)	<0.001
Drinking	Never	1.00 (reference)	
	Ex-drinker	0.97 (0.94, 1.00)	0.048
	Current drinker	0.98 (0.95, 1.00)	0.049
Physical activity	Bad	1.00 (reference)	
	Good^2^	1.03 (1.01, 1.04)	0.006
CVD risk factors^3^	0	1.00 (reference)	
	1	0.97 (0.95, 0.99)	0.002
	2	1.02 (1.00, 1.05)	0.087
	3	1.08 (1.04, 1.12)	<0.001
	4	1.20 (1.11, 1.29)	<0.001

aOR, adjusted odds ratio; CI, confidence interval; CVD, cardiovascular disease.

1 Adjusted for age, gender, education, smoking status, drinking status, physical activity, and CVD risk factors.

2 At least 150 minutes of moderate-intensity physical activity or 75 minutes of vigorous-intensity physical activity throughout the week.

3 CVD risk factors included hypertension, diabetes mellitus, dyslipidemia, and body mass index ≥25 kg/m^2^.

## SUPPLEMENTARY MATERIALS

Korean version is available at http://www.e-epih.org/.
