# Supplementary material for: Factors affecting awareness of myocardial infarction symptoms among the general public in Korea
Source: Epidemiol Health. 2020 May 18;42:e2020032. doi: 10.4178/epih.e2020032 (PMC7340619; doi:10.4178/epih.e2020032)
Supplement: Supplementary file 1 [file epih-42-e2020032-suppl.pdf]

Volume: 42, Article ID: e2020032

https://doi.org/10.4178/epih.e2020032

Factors Affecting Awareness of Myocardial Infarction Symptoms among the General Public

Kyong Sil Park

School of Nursing, Cheju Halla University, Jeju, South Korea

ABSTRACT

OBJECTIVES: 2017년도 지역사회건강조사 자료를 이용하여 일반인들의 심근경색증 증상의 인지도를 확인하고 심근경색증의 증상 인지도에 영향을 미치는 요인을 확인하고자 하였다.

METHODS: 지역사회건강조사 자료를 활용한 단면적 조사연구이다. 심근경색증 증상 5문항을 바탕으로 인지군(awareness group) (5가지 증상 모두 '예'로 응답한 경우), 비인지군(unawareness group) (5가지 중 1가지라도 '아니오' 또는 '모름'으로 응답한 경우)으로 구분하여 분석하였다.

RESULTS: 총 228,281명의 대상자 중에서 한국의 심근경색증 증상 인지율은 42.4%이다. 흉통 및 호흡곤란 증상은 높은 인지율을, 팔, 어깨, 턱, 목, 등의 통증 및 위장관계 증상은 낮은 인지율을 보였다. 여성이 남성에 비해 전반적인 인지도가 높았으나, 흉통 및 흉부 불편감은 더 낮은 인지도을 보였다. 심근경색증 증상 인지도에 영향을 미치는 요인은 성별, 연령, 교육수준, 직업, 흡연, 음주, 신체비활동, 심혈관질환 위험 요인이 확인되었다.

CONCLUSIONS: 일반인들의 심근경색증 증상의 인지도를 제고하기 위해 성별, 연령, 교육수준, 직업을 고려하여 교육 및 홍보를 실시해야 한다. 그리고, 전형적인 심근경색증 증상뿐만 아니라 비전형적인 심근경색증 증상에 대한 인식에 대한 정부, 교육기관, 의료기관 등의 적극적인 노력이 필요하다. 또한, 금연, 금주, 신체활동 증진에 대한 보건정책과 심혈관질환 위험요인을 가진 대상자들의 지속적인 모니터링 및 관리가 필요하다.

KEY WORDS: Myocardial infarction symptom, Heart attack symptom, Awareness, Knowledge, Korean

Volume: 42, Article ID: e2020032

https://doi.org/10.4178/epih.e2020032

INTRODUCTION

국내 심장질환(heart diseases)의 사망률은 2018년 기준 인구 10만 명당 62.4명으로 2008년 기준 인구 10만 명당 43.4명에 비해 지속적인 증가 추세이며, 특히 심근경색증 및 협심증 등 허혈성 심장질환(ischemic heart disease, IHD)의 사망률이 30.9명으로 심장질환 사망률의 약 50%를 차지한다 [1]. 뇌혈관질환(cerebrovascular diseases)으로 인한 사망률은 감소하는 추세이나, 심장질환으로 인한 사망률은 지속적으로 증가하고 있어[1] 사회경제적 질병부담이 크다. 심근경색증은 심장의 관상동맥이 혈전에 의해 완전히 막혀 심장 근육이 괴사되는 질환으로 심부전, 부정맥 등의 합병증, 장애, 사망을 초래할 수 있으므로 심근경색증 발생 직후 빠른 대처와 치료는 환자 예후를 결정하는 가장 중요한 요인이 된다[2].

2017년 일반인의 급성심근경색 증상 인지율은 45.5%이며[3], 심뇌혈관질환의 증상발현 후 골든 타임이라고 정의되는 3시간 이내에 병원에 도착하는 환자는 47.7%에 불과하여[4] 심장질환의 인지도와 초기 대응이 여전히 미흡한 수준이다. 심장질환의 조기 대처 능력을 향상하기 위해서 심장질환 및 선행질환을 가지고 있는 대상자뿐만 아니라 지역사회내에서 살고 있는 일반인들이 심장질환 발생 시 골든타임 내 적절한 치료를 받을 수 있도록 평상시 증상과 대처방법에 대한 사전 인지가 중요하다[3].

심근경색증 환자가 처음 증상을 느끼고 병원에 찾기까지 소요된 치료추구시간의 경우 남성은 평균 13시간, 여성은 20시간이며, 여성은 남성보다 증상에 대한 소극적인 행동 및 태도를 보였다 [5]. 또한 급성심근경색의 증상에서 남성은 흉통, 발한 등의 전형적인 증상을 호소하는 반면에 여성은 등, 턱, 목에 오는 통증, 상복부 불편감, 호흡곤란, 피로, 식욕부진 등의 비전형적인 증상을 호소하였다[5-7]. 특히, 한국 심근경색증 환자들은 자신의 통증의 원인을 심장이 아닌 '소화불량 또는 위장관 문제'로 잘못 인지하고 있었다[5]. 일반인의 낮은 인지율, 심근경색증 환자의 성별에 따른 다른 인식과 태도는 환자들의 치료추구시간의 지연을 발생시켜 골든타임 내 적절한 치료를 받을 수 없게 만드는 요인으로 작용할 것이다. 심근경색증 환자 발생 시 빠른 대처를 할 수 있도록 지역사회 내의 일반인들의 인지율을 파악하고 올바른 치료추구행동을 하고 있는지에 대한 조사를 할 필요가 있다.

심근경색증 증상의 인지도에 영향을 미치는 요인으로 성별, 연령, 인종, 교육수준, 수입, 직업, 만성질환, 흡연, 음주, 신체활동, 이웃의 심근경색증 경험, 심근경색증 지식, 홍보물 접촉 등이 보고되고 있다[8-19]. 한국인의 인식도에 대한 연구가 진행되고 있으나, 제한된 지역에서 적은 대상자로 연구가 진행되었거나, 주로 사회경제적 요인만을 확인하여 심혈관질환에 영향을 미치는 행동적 위험 요인, 대사 위험 요인 등을 고려하지 않았기 때문에 한계성이 있다.

따라서 대규모 지역사회 기반으로 조사된 자료를 이용하여 심근경색증 증상 5문항을 바탕으로 일반인들의 심근경색증 증상의 인지도를 확인하고 심근경색증의 증상 인지도에 영향을 미치는 요인을 확인하고자 한다.

Volume: 42, Article ID: e2020032

https://doi.org/10.4178/epih.e2020032

MATERIALS AND METHODS

Research design

이 연구는 성별에 따라 일반인의 심근경색증 증상 인지도와 인지도에 영향을 미치는 요인을 확인하기 위해 2017년도 지역사회건강조사(Community Health Survey, CHS) 자료를 이용한 단면적 조사연구이다.

Study population

연구대상자는 2017년도 지역사회건강조사 원시자료를 활용하였다. 원시자료 228,381명의 대상자 중 심근경색증 증상 문항에서 '응답거부'로 표기한 100명은 대상자 선정에서 제외하였다. 따라서, 최종 228,281명을 연구대상자로 선정하였다.

Ethics statement

연구자료는 지역사회건강조사 자료를 활용한 연구이므로 윤리적인 승인이 필요하지 않고, 지역사회건강조사 웹 사이트를 통해 신청을 하여 승인받은 후 지역사회건강조사로부터 대상자의 개인 정보가 삭제된 원시자료를 받았다.

Measurements

대상자의 인구사회학적 특성으로 성별, 연령, 교육수준, 소득수준, 결혼상태, 직업, 월 가구 소득, 거주지이다. 직업은 전문행정관리 및 사무직(관리자, 전문가 및 관련종사자, 사무종사자), 판매서비스직 및 기능단순노무직(서비스종사자, 판매종사자, 농림어업종사자, 기능원 및 관련기능종사자, 장치, 기계조작 및 조립종사자, 단순노무종사자, 군인), 무직 및 주부(무직, 주부, 학생)로 범주화하였다. 심혈관질환 관련 선행질환 중 고혈압, 당뇨, 이상지질혈증은 '의사의 진단 여부' 또는 '현재 치료 여부'에 '예'로 응답한 경우 질환이 있는 것으로 구분하였다.

심근경색증 증상의 인식은 "심근경색증의 증상이라고 생각하는 문항은?" : 1) 턱, 목, 등에 통증이나 답답함, 2) 힘이 없으며, 어지럽고, 울렁거리거나 식은땀, 3) 가슴에 통증이나 압박감 또는 짓누르는 느낌, 4) 갑자기 팔, 어깨에 통증이나 불편감, 5) 갑자기 숨이 참으로 구성되어 있으며, '예' 또는 '아니오'로 응답하도록 하였다. 심근경색증 증상 문항에서 '모름'으로 응답한 경우는 심근경색증에 대한 지식을 모른다고 판단하여 '아니오'로 재코딩하였다. '응답거부'로 응답한 경우는 결측 처리하여 대상자선정에서 제외하였다. 심근경색증 5가지 증상은 미국 질병통제예방센터(Centers for Disease Control and Prevention, CDC) 에서 제시하는 심근경색증의 주요 증상 5가지로[20], 모두 '예'로 응답한 대상자는 '인지군(awareness group)'으로, 5가지 중 1가지라도 '아니오'로 응답한 대상자는 '비인지군(unawareness group)'으로 배정하였다[9-10, 14].

Data analysis

자료분석은 SPSS program version 20을 이용하였다. 명목변수는 사례수와 백분율로, 연속변수는 평균 및 표준 편차로 제시하였다. 심근경색증 증상의 인지도를 확인하기 위해 카이제곱 검정을 실시하였고, 비인지군에 영향을 미치는 요인을 확인하기 위해서 다중 로지스틱 회귀분석(Multivariate

Volume: 42, Article ID: e2020032

https://doi.org/10.4178/epih.e2020032

logistic regression)을 실시하였다. 통계적 유의성은 p <.05으로 정하였다.

RESULTS

인구사회학적 특성을 살펴보면 남성 44.9%, 여성 55.1%이며, 연령은 60세 이상이 39.2%로 높았다. 현재흡연자가 17.5%, 현재음주자가 67.2%였다. 신체활동이 나쁜 대상자는 66.2%이며, 고혈압 27.4%, 당뇨 11.0%, 이상지혈증 17.4%, BMI 25 ㎏/㎡이상인 대상자 27.4%였다(Table 1).

5가지 심근경색증 증상 중 1개도 인지하지 못한 대상자는 11.8%, 5개 모두 인지하는 대상자는 42.4%였다. 5개 증상의 인지율에 따라 분류된 인지군과 비인지군을 비교한 결과, 비인지군에서 5개 증상 중 1개도 인지하지 못한 대상자는 20.5%, 5개 증상 중 4개를 인지한 경우는 28.2%였다(Table 2).

심근경색증 대표적인 5가지 증상을 비교한 결과, '가슴에 통증이나 압박감 또는 짓누르는 느낌' 83.0%, '갑자기 숨이 참' 78.2%, '힘이 없으며, 어지럽고, 울렁거리거나 식은땀' 69.2%, '턱, 목, 등에 통증이나 답답함' 63.3%, '갑자기 팔, 어깨에 통증이나 불편감' 53.8% 순으로 인지율을 보였다. 여성이 남성에 비해 4개 증상에서 높은 인지율을 보였으나, '가슴에 통증이나 압박감 또는 짓누르는 느낌' 증상은 남성이 여성에 비해 인지율이 높았다. 연령은 5가지 증상 모두 40-59세 인지율이 높았으며, 60세 이상 인지율이 가장 낮았다. 교육 수준은 5가지 증상 모두 대졸자가 고졸자에 비해 더 높았다. 기혼자는 미혼자에 비해 5가지 증상 모두 인지율이 높았으며, 직업에서 전문행정관리 및 사무직이 가장 높은 인지율을, 무직 및 주부가 가장 낮은 인지율을 보였다. 월평균 가구소득 수준이 높을수록 각 증상의 인지율은 높았다. 흡연과 음주를 하는 대상자는 인지율이 낮았으며, 비흡연자 및 비음주자는 인지율이 높았다. 신체활동을 잘하는 대상자는 인지율이 높았다. 심혈관질환 위험 요인(고혈압, 당뇨, 이상지질혈증, 비만)을 모두 가지고 있는 대상자는 5가지 증상의 인지율이 가장 높았다(Table 3).

심근경색증 발생 시 예상되는 치료추구행동을 비교한 결과, '119 전화'가 인지군과 비인지군 각각 85.6%, 81.3%로 높은 치료추구행동을 보였다(Table 4).

심근경색증 증상의 인지도에 영향을 미치는 인구사회학적 및 건강 관련 요인을 확인하기 위해 multivariate logistic regression 한 결과, 여성(odds ratio[OR] 1.03, 95% confidence interval [CI] 1.01-1.06, p=0.010), 40-59세(OR 1.32, CI 1.29-1.35, p<.001), 대졸자(OR 1.31, CI 1.28-1.33, p<.001), 무직 및 주부(OR 0.81, CI 0.79-0.84, p<.001), 현재흡연자(OR 0.84, CI 0.82-0.86, p<.001), 현재음주자(OR 0.98, CI 0.95-1.00, p=0.049), 주(week)에 중등도 신체활동 150분 또는 고강도 신체활동 75분 이상하는 대상자(OR 1.03, CI 1.01-1.04, p=0.006), 심혈관질환 위험요인 4개 모두 가진 대상자(OR 1.20, CI 1.11-1.29, p<.001)는 급성심근경색증 증상 인지도에 영향을 미치는 요인으로 확인되었다(Table 5).

Volume: 42, Article ID: e2020032

https://doi.org/10.4178/epih.e2020032

DISCUSSION

심근경색증 증상 발생 시 골든타임 내 적절한 응급대처를 위한 필수적인 요소는 심근경색증의 증상에 대해 잘 알고 있는 것이다[3]. 연구결과, 심근경색증 증상 5가지 중 1개도 인지하지 못하는 대상자가 11.8%, 증상 5가지 모두 인지하고 있는 고인지군의 대상자는 42.4%였다. 2010년 국내 연구의 심근경색증 인지율(7.1%[9], 10.9%[10])과 비교하면, 현재의 심근경색증 인지율은 42.4%로 매우 상승하였다. 이는 2008년부터 권역심뇌혈관질환센터를 지정하여 조기증상교육 및 홍보활동을[4] 지속적으로 해온 결과로 볼 수 있다. 하지만, 미국의 인지율 53.0%에 비해[13] 한국의 인지율은 상대적으로 낮아 인지율을 제고할 수 있는 관리방안이 필요하겠다. 한국인들의 심근경색증 증상에 대한 올바른 인식이 정착된다면 심근경색증 발생 시 골든타임 내 적절한 대처를 받을 수 있을 뿐 아니라 사회경제적 질병부담 감소에도 큰 이득이 있을 것으로 기대된다.

이 연구대상자들은 심근경색증의 대표적인 5가지 증상 중 ‘팔, 어깨에 통증이나 불편감’, ‘턱, 목, 등에 통증이나 답답함’ 증상을 심근경색증의 증상으로 인지하지 못하는 사람이 가장 많았으며 ‘가슴에 통증이나 압박감 또는 짓누르는 느낌’ 증상은 심근경색증의 증상으로 잘 인지하고 있었다. 2010년 한국 연구결과에 비해[9-10] 전체적인 인지율은 상승하였으나, 각 증상에 대한 인지율은 여전히 낮은 상태이다. 즉, 흉통은 심근경색증의 전형적인 증상으로 인지하고 있으나 팔, 어깨, 턱, 목, 등으로 퍼지는 방사통의 형태의 증상은 53.8%로 인지율이 낮았다. 국외의 경우[13] ‘팔, 어깨에 통증이나 불편감’의 인지율이 85.7%로 한국과 상당한 인식의 격차를 보이고 있다. 국외의 심근경색증 증상 5가지가 대체적으로 고루 높은 인지를 보이는 반면[13], 한국은 흉통과 호흡곤란을 제외한 나머지 증상에 대한 인지는 낮았다. 따라서, 우리가 잘 알고 있는 심근경색증 증상 외에 잘 인지하지 못하는 증상에 포커스하여 교육 및 홍보를 시행해야 하겠다.

심근경색증 증상 인지도에 예측 요인으로 성별이 확인되었다. 심근경색증 증상의 전체적인 인지율은 여성이 남성보다 높은 반면, ‘가슴에 통증이나 압박감 또는 짓누르는 느낌’의 증상은 여성보다 남성에서 인지율이 더 높았다. 국외의 경우[14] 심근경색증의 주요 5가지 증상 모두 여성의 인지율이 높음을 고려하면 한국에서 일반인 남성에게 심근경색증의 증상으로 가슴통증 및 불편감이 대표적인 증상으로 인식된다는 것을 예상할 수 있다. 이 결과는 오히려 다른 심근경색증 증상이 발생했을 때, 특히 자신의 여성 가족에게 비전형적인 심근경색증 증상이 발생하였을 때, 심혈관질환의 예방과 치료를 놓칠 수 있는 가능성이 높아진다는 해석이 가능하다. 따라서, 일반인 남성을 대상으로 전형적인 심근경색증의 증상 외에 다양한 심근경색증 증상을 교육할 필요가 있다.

심근경색증 증상 인지도의 주요 예측 요인으로, 다른 연령군에 비해 40-59세 중년의 인지도가 높음을 확인하였다. 40-59세 중년의 경우 심근경색증 발생률이 높아[17] 질환에 따른 정보 노출이 많아 증상에 대한 인지율이 높다. 그러나 19-39세 젊은 성인의 경우 활발한 경제활동 시기로 시간의 부족과 심근경색증 관리에 대한 필요성의 부족으로[21] 실질적인 증상 인식이 어려웠다. 또한, 60세 이상은 노화로 인하여 심근경색증 증상을 이해하는 측면이 부족하여[5] 연령군에 따라 다른

Volume: 42, Article ID: e2020032

https://doi.org/10.4178/epih.e2020032

인지도를 보인다고 판단된다.

교육수준, 직업 등의 사회 경제적 지위(socio-economic status, SES)가 높은 대상자는 심근경색증 증상 인지도가 높았다. 선행 연구에서도 교육수준, 직업이 심근경색증 인지도와 연관성이 있었다 [8-19]. 사회 경제적 지위가 높은 사람들은 적극적으로 정보를 수용하고 실천할 수 있는 유리한 조건이 있다. 반면에 사회 경제적 지위가 낮은 사람들은 정보의 제약이 있거나 불리한 조건들이 있어[22] 인지도에 영향을 미치는 것으로 판단된다. 따라서, 사회 경제적 지위에 따른 건강불평등을 줄이기 위해 교육수준이 낮고, 판매서비스직 및 기능단순노무직, 무직 및 주부를 중심으로 그들의 수준에 맞는 홍보 및 교육을 실시하는 것이 필요하다. 젊은 집단 및 노년 집단을 중심으로 다양한 매체를 활용하여 심근경색증 증상 인지율을 높이는 방안이 필요하다. 특히, 20-30대는 인터넷 활용도가 높으며, 60대 이상은 보건기관 이용 시 인지도를 향상시킬 수 있으므로[11] 다양한 방법을 통해 효율적인 홍보 및 교육을 실시해야 하겠다.

심혈관질환의 위험요인인 흡연, 음주, 신체비활동은 심근경색증 증상 인지도에 영향요인으로 확인되었다. 흡연 또는 음주를 한 사람은 심근경색증 증상 인지도가 낮았다. 선행 연구에서 흡연과 폭음은 심혈관질환 지식과 연관성이 높았다[11-12, 19]. 건강을 해치는 행위인 흡연, 음주는 건강문제에 대한 전반적 인식 부족에서 기인하며, 건강문제에 대한 전체적 인식 부족으로 심근경색증 증상 인지도 낮은 것으로 판단된다. 주(week)에 중등도 신체활동 150분 또는 고강도 신체활동 75분 이상하는 대상자의 심근경색증 증상 인지도가 높았다. 신체활동과 심근경색증 인지도의 연관성 연구가 드물지만, 최근에 규칙적인 운동과 심근경색증 인지도와 연관성이 있다는 연구결과[12]가 있었다. 흡연, 음주, 신체비활동은 심혈관질환을 일으키는 행동적 위험요인이면서[23] 심혈관질환의 증상 인지에도 연관성이 있음을 확인하였다. 건강에 대한 인식의 변화가 건강행위를 변화시키는 주요 요인이므로[24] 심근경색증 증상 인지도 및 흡연, 음주, 신체비활동 등의 건강행태도 함께 증진시킬 수 있는 관심과 노력이 필요하다.

고혈압, 당뇨, 이상지질혈증, 비만 중 3개 이상 심혈관질환 위험요인을 가지고 있는 경우 심근경색증 증상 인지도는 높았다. 위험요인을 많이 가지고 있는 사람은 이 위험요인들이 심혈관질환의 대표적 위험질환임을 인지하고 있기 때문에 심근경색증 증상 인지도가 높은 것으로 파악된다. 이에 반해 위험요인을 0-2개 가지고 있는 대상자는 오히려 인지도가 낮거나 유의하지 않았다. 이것은 고혈압, 이상지질혈증이 있는 대상자는 인지도가 높고, 당뇨, 비만이 있는 대상자는 유의하지 않거나 인지도가 낮은 선행연구[11-12]를 통해 해석이 가능하다. 당뇨와 비만이 있는 대상자는 심근경색증 증상 인지도가 낮아 위험요인의 개수에 영향을 미쳤을 것으로 유추된다. 하지만, 정확한 검증을 위해 추후 심혈관질환의 대표 위험요인과 심근경색증 증상 인지도의 연관성 연구가 필요하다.

이 연구는 심근경색증 환자가 아닌 일반인들을 대상으로 심근경색증의 대표적인 5가지 증상에 따른 차이를 확인, 인지도에 영향을 미치는 요인들을 파악함으로써 심근경색증 조기 증상 인지 및 대처방법 향상을 위한 교육적 중재자료로 제공될 수 있다는 의의가 있다. 또한, 대표성을

가지고 있는 자료를 활용하였기 때문에 연구결과를 전 국민에게 일반화할 수 있다는 장점이 있다. 하지만, 일 시점의 자기보고식 설문에 의한 단면적 조사연구이므로 인과관계를 확인할 수 없고, 결측 자료를 제외하였기 때문에 제한점을 가지고 있다.

한국에서 일반인들의 심근경색증 증상의 인지도를 제고하기 위해 성별, 연령, 교육수준, 직업을 고려하여 교육 및 홍보를 실시해야 한다. 그리고, 전형적인 심근경색증 증상뿐만 아니라 비전형적인 심근경색증 증상에 대한 인식에 대한 정부, 교육기관, 의료기관 등의 적극적인 노력이 필요하다. 또한, 금연, 금주, 신체활동 증진에 대한 보건정책적 관리방안과 심혈관질환 위험요인이 많은 대상자들의 지속적인 추적관찰 및 관리도 필요하다. 이러한 노력을 통해 심근경색증 증상 인지율이 향상되고 심근경색증 환자의 치료추구시간이 단축되어, 환자의 삶의 질 향상, 사회경제적 질병부담 감소 및 공공 보건정책에 긍정적 영향을 줄 수 있을 것이다.

CONFLICT OF INTEREST

본 논문의 저자는 어떠한 이해관계도 없음을 밝힙니다.

REFERENCE

1. Statistical Korea. The cause of Death Statistics; 2018 [cited 2020 Jan 18] Available from http://kostat.go.kr/portal/korea/kor_nw/1/6/1/index.board?bmode=read&bSeq=&aSeq=377606&pageNo=1&rowNum=10&navCount=10&currPg=&searchInfo=&sTarget=title&sTxt=

2. Oh HS, Jang KS, Jeong MH. Decision tree model of treatment-seeking delay among patients with acute coronary syndrome. Korean Journal of Health Promotion and Disease Prevention 2008;8:235-244.

3. Ministry of Health & Welfare. Cardiovascular Disease Management Comprehensive Plan; 2018 [cited 2020 Jan 18] Available from http://www.mohw.go.kr/react/al/sal0301vw.jsp?PAR_MENU_ID=04&MENU_ID=0403&CONT_SEQ=345946&page=1

4. Ministry of Health & Welfare. Regional Cardiocerebrovascular Center Operating Instructions; 2019 [cited 2020 Jan 18] Available from http://www.mohw.go.kr/react/jb/sjb030301vw.jsp?PAR_MENU_ID=03&MENU_ID=032903&CONT_SEQ=349417&page=1

5. Won MS, Shin NM, Kim E. Gender Differences in Delay Seeking Treatment and Related Experiences in Patients with Acute Myocardial Infarction. Korean Journal of Adult Nursing 2016;28:459-469.

6. Coventry LL, Finn J, Bremner AP. Sex differences in symptom presentation in acute myocardial infarction: a systematic review and meta-analysis. Heart & Lung 2011;40:477-491.

7. Seol SY, Jeong MH, Lee SH, Sohn SJ, Cho JY, Kim MC, et al. Impact of Gender Differences in Elderly Patients with Acute Myocardial Infarction. Korean Journal of Medicine 2019;94:96-106.

8. Kim EM, Hwang SY, Kim AL. Knowledge of stroke and heart attack symptoms and risk factors among rural elderly people: a questionnaire survey. Korean circulation journal 2011;41:259-264.

9. Na YJ, Lee YM, Kim KY, Choi YH. Awareness of Myocardial Infarction Warning Symptoms and Related Factors among Residents in a Province. J Health Info Stat 2015;40:32-43.

10. Kim HS, Lee H, Kim K, Park HK, Park KS, Kang GW, et al. The general public’s awareness of early symptoms of and emergency responses to acute myocardial infarction and related factors in south korea: a national public telephone survey. Journal of epidemiology 2016;26:233-241.

11. Lee YH, Noh SE. Factors Related to Awareness of Cardio-cerebrovascular Disease among Korean Adults: the 2013 Community Health Survey. Korean J Health Promot 2017;17:99-108.

12. Han CH, Kim H, Lee S, Chung JH. Knowledge and Poor Understanding Factors of Stroke and Heart Attack Symptoms. International Journal of Environmental Research and Public Health 2019;16:E3665.

13. Mahajan S, Valero-Elizondo J, Khera R, Desai NR, Blankstein R, Blaha MJ, et al. Variation and Disparities in Awareness of Myocardial Infarction Symptoms Among Adults in the United States. JAMA Network Open 2019;2:e1917885.

https://doi.org/10.4178/epih.e2020032

14. Fang J, Gillespie C, Keenan NL, Greenlund KJ. Awareness of heart attack symptoms among US adults in 2007, and changes in awareness from 2001 to 2007. Future cardiology 2011;7:311-320.

15. Chau PH, Moe G, Lee SY, Woo J, Leung AY, Chow CM, et al. Low level of knowledge of heart attack symptoms and inappropriate anticipated treatment-seeking behaviour among older Chinese: a cross-sectional survey. J Epidemiol Community Health 2018;72:645-652.

16. Swanoski MT, Lutfiyya MN, Amaro ML, Akers MF, Huot KL. Knowledge of heart attack and stroke symptomology: a cross-sectional comparison of rural and non-rural US adults. BMC public health 2012;12:283.

17. Holly Felix MRN, Rowland B, Long CR, Bursac Z, McElfish PA. Level of Recommended Heart Attack Knowledge among Native Hawaiian and Pacific Islander Adults in the United States. Hawai'i Journal of Medicine & Public Health 2019;78:61.

18. Lutfiyya MN, Bardales R, Bales R, Aguero C, Brady S, Tobar A, Lipsky MS. Awareness of heart attack and stroke symptoms among Hispanic male adults living in the United States. Journal of immigrant and minority health 2010;12:761-768.

19. Aminde LN, Takah N, Ngwasiri C, Noubiap JJ, Tindong M, Dzudie A, et al. Population awareness of cardiovascular disease and its risk factors in Buea, Cameroon. BMC Public Health 2017;17:545.

20. Centers for Disease Control and Prevention. Heart Attack; 2019 [cited 2020 Mar 19] Available from https://www.cdc.gov/heartdisease/heart_attack.htm

21. Kim CG, Lee SH, Cha SK. Influencing Factors on Cardio-cerebrovascular Disease Risk Factors in Young Men: Focusing on Obesity Indices. Journal of Korean Nursing Science 2017;19:1-10.

22. Kim JY. The Relationship between Socioeconomic Status and Health in Korea - Focusing on Age Variations. Korean Journal of Sociology 2007;41:127-153.

23. World Health Organization. Global atlas on cardiovascular disease prevention and control; 2011 [cited 2020 Mar 19] Available from https://www.who.int/cardiovascular_diseases/publications/atlas_cvd/en/

24. Choi JS. The effect of early detection of hypertension and diabetes on smoking and alcohol drinking. Health Soc Welf Rev 2007;27:103-130.

Volume: 42, Article ID: e2020032

https://doi.org/10.4178/epih.e2020032

Table 1. Socio-demographic and health related characteristics (n=228,281)

Characteristics	Category	N (%)
Gender	Male	102,431 (44.9)
	Female	125,850 (55.1)
Age (years)	19-39	54,203 (23.7)
	40-59	84,600 (37.1)
	≥60	89,478 (39.2)
Education	High school	158,978 (69.6)
	≥College	69,303 (30.4)
Marital status	Married	192,864 (84.6)
	Unmarried/divorced/widowed	35,167 (15.4)
Occupation	Professional/manager/clerk	44,952 (19.7)
	Sales/service/manual	99,390 (43.6)
	Unemployed/housewives	83,674 (36.7)
Monthly household income (10,000 Won)	<200	81,663 (36.1)
	200-399	74,647 (33.0)
	≥400	69,898 (30.9)
Residential area	Urban	109,362 (47.9)
	Rural	118,919 (52.1)
Smoking	Current smoker	39,965 (17.5)
	Ex-smoker	42,041 (18.4)
	Never	146,268 (64.1)
Drinking	Current drinker	153,441 (67.2)
	Ex-drinker	32,887 (14.4)
	Never	41,940 (18.4)
Physical activity	Bad	150,977 (66.2)
	Good[1]	77,191 (33.8)
CVD risk factors	Hypertension	62,500 (27.4)
	Diabetes mellitus	25,200 (11.0)
	Dyslipidemia	39,775 (17.4)
	BMI≥25 kg/m²	60,037 (27.4)

CVD, cardiovascular disease; BMI, body mass index
[1]Do at least 150 minutes of moderate-intensity physical activity or 75 minutes of vigorous-intensity physical activity throughout the week.

Volume: 42, Article ID: e2020032

https://doi.org/10.4178/epih.e2020032

Table 2. Awareness of myocardial infarction symptom

Category	Total (n=228,281) N(%)	Awareness (n=96,783) N(%)	Unawareness (n=131,498) N(%)
None	26,985 (11.8)	0 (0.0)	26,985 (20.5)
1	9,822 (4.3)	0 (0.0)	9,822 (7.5)
2	21,592 (9.5)	0 (0.0)	21,592 (16.4)
3	35,975 (15.8)	0 (0.0)	35,975 (27.4)
4	37,124 (16.3)	0 (0.0)	37,124 (28.2)
5	96,783 (42.4)	96,783 (100.0)	0 (0.0)

$p < .001$

Volume: 42, Article ID: e2020032

https://doi.org/10.4178/epih.e2020032

Table 3. Awareness of each symptom of myocardial infarction (n=228,281)

Characteristics	Category	Pain or discomfort in the jaw, neck, or back	Feeling weak, light-headed, or faint, cold sweat	Chest pain or pressure, squeezing	Pain or discomfort in one or both arms or shoulders	Shortness of breath
		% (*p*)	% (*p*)	% (*p*)	% (*p*)	% (*p*)
Total		63.3	69.2	83.0	53.8	78.2
Gender	Male	62.3	68.6	83.2	53.2	77.5
	Female	64.1	69.7	82.8	54.3	78.8
		(<0.001)	*(<0.001)*	(0.013)	*(<0.001)*	*(<0.001)*
Age (years)	19-39	62.8	67.4	84.4	52.7	78.5
	40-59	67.7	73.6	88.3	57.8	82.9
	≥60	59.4	66.2	77.1	50.7	73.5
		(<0.001)	*(<0.001)*	*(<0.001)*	*(<0.001)*	*(<0.001)*
Education	High school	60.8	67.5	80.5	51.9	76.2
	≥College	68.9	73.2	88.8	58.2	82.8
		(<0.001)	*(<0.001)*	*(<0.001)*	*(<0.001)*	*(<0.001)*
Marital status	Married	64.0	70.0	83.1	54.5	78.6
	Unmarried/divorced/widowed	59.4	65.3	82.3	50.0	76.0
		(<0.001)	*(<0.001)*	*(<0.001)*	*(<0.001)*	*(<0.001)*
Occupation	Professional/manager/clerk	69.7	73.7	89.2	58.8	83.1
	Sales/service/manual	63.2	69.6	83.3	54.0	78.7
	Unemployed/housewives	60.0	66.4	79.3	51.0	75.0
		(<0.001)	*(<0.001)*	*(<0.001)*	*(<0.001)*	*(<0.001)*
Monthly household income (10,000 Won)	<200	58.7	65.3	76.8	49.9	73.1
	200-399	65.0	71.0	85.5	55.7	80.3
	≥400	67.0	72.1	87.7	56.6	82.0
		(<0.001)	*(<0.001)*	*(<0.001)*	*(<0.001)*	*(<0.001)*
Residential area	Urban	62.9	68.5	83.8	53.5	78.6
	Rural	63.7	69.9	82.2	54.1	77.8
		(<0.001)	*(<0.001)*	*(<0.001)*	*0.006*	*(<0.001)*
Smoking	Current smoker	60.4	67.1	82.4	51.4	76.6
	Ex-smoker	63.1	69.4	83.4	53.8	77.9
	Never	64.1	69.8	83.0	54.5	78.7
		(<0.001)	*(<0.001)*	*(<0.001)*	*(<0.001)*	*(<0.001)*
Drinking	Current drinker	64.5	70.2	85.2	54.5	79.9
	Ex-drinker	61.6	67.9	80.5	52.8	76.3
	Never	60.2	66.8	77.0	52.3	73.5
		(<0.001)	*(<0.001)*	*(<0.001)*	*(<0.001)*	*(<0.001)*
Physical activity	Bad	62.4	68.3	82.0	53.4	77.3
	Good[1]	65.0	71.0	85.0	54.6	79.9
		(<0.001)	*(<0.001)*	*(<0.001)*	*(<0.001)*	*(<0.001)*
CVD risk factors[2]	0	64.0	69.6	83.9	54.4	79.1
	1	62.2	68.2	82.0	52.7	77.0
	2	62.5	69.1	81.8	53.7	77.4

Volume: 42, Article ID: e2020032

https://doi.org/10.4178/epih.e2020032

3	64.1	70.9	82.7	54.2	78.4
4	65.3	72.2	83.9	56.8	79.3
	(0.003)	(0.118)	*(<0.001)*	(0.167)	*(<0.001)*

CVD, cardiovascular disease; BMI, body mass index

[1]Do at least 150 minutes of moderate-intensity physical activity or 75 minutes of vigorous-intensity physical activity throughout the week.

[2]CVD risk factors were included Hypertension, Diabetes mellitus, Dyslipidemia, BMI≥25 ㎏/㎡

Volume: 42, Article ID: e2020032

https://doi.org/10.4178/epih.e2020032

Table 4. Expected treatment seeking behavior at myocardial infarction onset

Behaviors	Total (n=227,740)<br>N(%)	Awareness (n=96,764)<br>N(%)	Unawareness(n=130,976)<br>N(%)
Hospital	28,387 (12.5)	10,820 (11.2)	17,567 (13.4)
Oriental hospital	440 (0.2)	162 (0.2)	278 (0.2)
Call 119	189,385 (83.2)	82,849 (85.6)	106,536 (81.3)
Call family	7,979 (3.5)	2,397 (2.5)	5,582 (4.3)
Others	1,549 (0.7)	536 (0.6)	1,013 (0.8)

p <.001

Volume: 42, Article ID: e2020032

https://doi.org/10.4178/epih.e2020032

Table 5. Predictors of awareness of myocardial infarction symptoms

Characteristics	Category	aOR (95% CI)[1]	*p*
Gender	Male	ref	
	Female	1.03 (1.01-1.06)	0.010
Age (year)	19-39	ref	
	40-59	1.32 (1.29-1.35)	<*.001*
	≥60	1.06 (1.04-1.09)	<*.001*
Education	High school	ref	
	≥College	1.31 (1.28-1.33)	<*.001*
Occupation	Professional/manager/clerk	ref	
	Sales/service/manual	0.90 (0.88-0.93)	<*.001*
	Unemployed/housewives	0.81 (0.79-0.84)	<*.001*
Smoking	Never	ref	
	Ex-smoker	0.96 (0.93-0.99)	0.006
	Current smoker	0.84 (0.82-0.86)	<*.001*
Drinking	Never	ref	
	Ex-drinker	0.97 (0.94-1.00)	0.048
	Current drinker	0.98 (0.95-1.00)	0.049
Physical activity	Bad	ref	
	Good[2]	1.03 (1.01-1.04)	0.006
CVD risk factors[3]	0	ref	
	1	0.97 (0.95-0.99)	0.002
	2	1.02 (1.00-1.05)	0.087
	3	1.08 (1.04-1.12)	<*.001*
	4	1.20 (1.11-1.29)	<*.001*

aOR, adjusted odds ratio; CI, confidence interval; CVD, cardiovascular disease.

[1]Adjusted for age, gender, Education, Smoking, Drinking, physical activity, CVD risk factors.

[2]Do at least 150 minutes of moderate-intensity physical activity or 75 minutes of vigorous-intensity physical activity throughout the week.

[3]CVD risk factors were included Hypertension, Diabetes mellitus, Dyslipidemia, BMI≥25 ㎏/㎡
